# The prevalence of *Francisella* spp. in different natural surface water samples collected from northwest of Iran

**Published:** 2019-02

**Authors:** Mahdi Rohani, Abdolrazagh Hashemi Shahraki, Ahmad Ghasemi, Saber Esmaeili, Aynur Karadenizli, Ehsan Mostafavi

**Affiliations:** 1 Department of Microbiology, Pasteur Institute of Iran, Tehran, Iran; 2 National Reference Laboratory for Plague, Tularemia and Q Fever, Research Centre for Emerging and Reemerging Infectious Diseases, Pasteur Institute of Iran, Akanlu, Kabudar Ahang, Hamadan, Iran; 3 Department of Epidemiology and Biostatistics, Research Centre for Emerging and Reemerging Infectious Diseases, Pasteur Institute of Iran, Tehran, Iran; 4 Department of Bacteriology, Faculty of Medical Sciences, Tarbiat Modares University, Tehran, Iran; 5 Department of Medical Microbiology, Kocaeli University Medical School, Kocaeli, Turkey

**Keywords:** Tularemia, Environmental sample, Real-time polymerase chain reaction, *Francisella*

## Abstract

**Background and Objectives:**

*Francisella tularensis* has a wide distribution in northern hemisphere of the world. Up to now, there was little information about the *Francisella* spp. situation in the environmental samples in Iran. In this study we aimed to determine the prevalence of *Francisella* spp. in the environmental samples in northwest of Iran.

**Materials and Methods:**

A total of 237 natural water samples from ponds, rivers, lakes, springs and other surface waters from north western provinces of Iran (Kurdistan and Western Azerbaijan) were collected from September to November 2015. All samples were cultured for *Francisella* and other bacterial species and Real Time TaqMan PCR was performed on the concentrated and DNA extracted samples. For detection of the presence of bacterial DNA in the samples, two different targets in the genome of *Francisella*, *ISFtu2* and *fopA* were used.

**Results:**

Among the tested surface water samples, 40 (17.09%; 95% CI: 12.67–22.33%) and 12 (5.13%; 95%CI: 2.81–8.56%) samples were positive for *ISFtu2* and *fopA* respectively. None of them was positive in culture.

**Conclusion:**

The prevalence of *Francisella* spp. in the environmental samples in the west of Iran is high and it is comparable with Turkey, Iran's neighboring country. Use of higher copy number genes or *IS* like *ISFtu2* could improve the detection of this organism in the environmental samples.

## INTRODUCTION

The genus *Francisella* is a Gram-negative aerobic coccobacillus and contains two species: *F. tularensis* and *F. philomiragia. F. tularensis* is the causative agent of tularemia. The infection dose of this bacterium is less than 10 microorganisms and according to this highly infectious property, this agent is classified as a dangerous pathogen (Category A, CDC) (1). Currently *F. tularensis* is divided into four subspecies: *tularensis* (nearctica), *holarctica* (palaearctica), *mediasiatica*, and *novicida*, which differ in their distribution and virulence in humans. *F. tularensis* subspecies tularensis (type A) is predominant in USA and *F. tularensis* subspecies holarctica (type B) is common type in Asia and Europe (2). *F. tularensis* is found throughout the Northern hemisphere; however some researchers believe that this agent is detectable in each parts of the earth from human samples to environmental samples (3). The main reservoirs of this agent are diverse in different areas, from rabbit and ticks in USA and aquatic rodents and environmental waters in other parts. Infection with *F. tularensis* is also reported in various species including carnivores, ungulates, marsupials, birds, amphibians, fish, and livestock (4). Transmission of tularemia occurs in various routes from direct contact with infected materials, digestion of untreated water or undercooked meat, animal or arthropod bites to inhalation of contaminated aerosol or dust (2). The mortality rate of this infection in untreated condition in type A is 10–40% and in type B is about 1% (1).

The history of the presence of *F. tularensis* in Iran comes back to 1973 that the first detection of antibodies against this agent was reported in cattle and sheep in the northwest and in a porcupine in the southeast of Iran (5). The first human case of tularemia was reported in 1980 in Marivan city in Kurdistan province (6) and recently the second case of tularemia were reported in 2017 from a village near Marivan city (7). In recent years, the antibody against tularemia was also reported in rodents in the southeast and west of Iran (8, 9). In a study in 2014 in Sistan and Baluchistan province, southeast of Iran, the seroprevalence of tularemia amongst butchers and slaughterhouse workers was 6.52% and in another study on high risk groups in Kurdistan province, west of Iran, prevalence of antibodies against tularemia was reported 14.4% (10, 11). In similar studies on high-risk populations, the seroprevalence rate was reported 0.3–6.3% in Turkey (12–14), and 15.5% in Azerbaijan (15).

Environmental water is considered as one of the most important sources of *F. tularensis* subspecies holarctica and various outbreaks are reported that are linked to sources of contaminated waters (16). Dark and cold water are suitable situation for survive of *F. tularensis* for months and some studies indicate that this agent cannot live in warm waters (17).

Due to the fact that tularemia is an endemic disease in Turkey (Iran's northwest neighbor) and several clinical cases of this disease are reported annually from that country (18), and because of the recent detection of tularemia antibodies in the human population of the Republic of Azerbaijan (Iran's northern neighbor) (15), and recent evidences of the circulation of the bacteria in Iran (8–11), taking into account the fact that there is no information respect to contamination of water samples to *Francisella* spp. in Iran, this study was conducted to evaluate the surface waters in two north western provinces, Kurdistan and West Azerbaijan.

## MATERIALS AND METHODS

### Water sampling.

In this study, 130 and 107 surface water samples from ponds, rivers, lakes, springs and other surface waters from Kurdistan and West Azerbaijan were collected from September to November 2015, respectively. The samples were collected from eight different districts in Kurdistan province (from Saghez, Baneh, Divandareh, Bijar, Sanandaj, Dehgolan, Sarvabad and Marivan) and 11 different districts in West Azerbaijan province (from Maku, Urmia city, Mahabad, Shahin Dezh, Bukan, Sardasht, Piranshahr, Oshnavieh, Chaldoran, Salmas and Khoy). In each district about 2.5l liter water was collected from surface waters with mod and soil. For better investigation, the sampling was done from north, east, west, south and central region of each district. All samples were sent to the National Reference Laboratory for Plague, Tularemia and Q fever in Research Centre of Emerging and Reemerging Infectious Diseases of Pasteur Institute of Iran, under 4°C in less than 48 hours after sampling.

### Culture of water samples for *Francisella* spp.

Each sample was filtered and the paper filters were cultured on Cysteine Heart Agar with supplementation of 9% chocolatized sheep blood with supplemented antibiotics (19) including 8 ×10^4^ U l^−1^ polymyxin B, 2.5 mg l^−1^ amphotericin B, 4 mg l^−1^ cefepime, 100 mg l^−1^ of cyclohexamide and 4 mg l^−1^ vancomycin. Each medium was incubated for at least 72 hours and monitored for 6 days at 37°C, in candle jar and humid atmosphere.

### DNA extraction and Real time PCR.

**Fig. 1.**
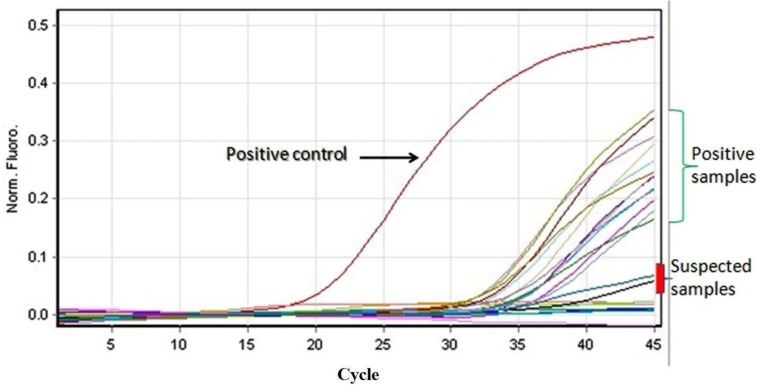
The positive reaction of real time PCR. The positive and suspected divisions have done based on Ct values of each reaction

For concentrating the samples, we used the vacuum pump and paper filter and for molecular detection, DNA was extracted from water samples using the soil DNA extraction kit (NucleoSpin^®^ Soil Kit-Germany), and the ISFtu2 real-time PCR was applied. The presence of the *fop-A* gene was examined in the samples that had positive results by ISFtu2 primers to confirm the presence of *Francisella* spp. (Table 1 and Fig. 1). The DNA of *F. tularensis* subsp *holarctica* NCTC 10857 was used as a positive control.

**Table 1.** Primers and probes used for laboratory testing

**Gene**	**Premieres**	**Sequences**	**Size (bp)**
*fopA*	*fopA*-F	AACAATGGCACCTAGTAATATTTCTGG	
	*fopA*-R	CCACCAAAGAACCATGTTAAACC	87
	Probe	FAM-TGGCAGAGCGGG	
		TACTAACATGATTGGT-TAMRA	

*ISFtu2*	*ISFtu2F*	TTGGTAGATCAGTTGGTAGGATAACC	97
	*ISFtu2R*	TGAGTTTTATCCTCTGACAACAATATTTC	
	Probe	FAM-AAAATCCATGCTATGACTGATGCTTTAGGTAATCCA-TAMRA	

## RESULTS

Among 237 tested water samples, 40 (17.09%; 95% CI: 12.67–22.33%) and 12 (5.13%; 95%CI: 2.81–8.56%) samples were positive for *ISFtu2* and *fopA* genes of *Francisella* spp., respectively (Table 2). None of them was positive in culture.

**Table 2.** The real-time PCR results of positive samples for *Francisella* spp.

**Province**	**District**	**No. collected**	**No. (%) positive by *ISFtu2***	**No. (%) positive by *fopA***
**Kurdistan**	Bijar	20	8 (40)	3 (15)
	Baneh	12	4 (33.33)	1 (8.33)
	Sanandaj	16	1 (6.25)	0 (0)
	Divandareh	14	1 (7.14)	0 (0)
	Saghez	22	0 (0)	0 (0)
	Dehgolan	12	1 (8.33)	0 (0)
	Sarvabad	16	2 (12.50)	1 (6.25)
	Marivan	18	0 (0)	0 (0)
	**Total**	**130**	**17 (13.08)**	**5 (3.85)**
**West Azerbaijan**	Maku	8	1 (12.50)	0 (0)
	Urmia	11	1 (9.09)	0 (0)
	Mahabad	14	2 (14.28)	0 (0)
	Shahin Dezh	11	0 (0)	0 (0)
	Bukan	9	1 (11.11)	1 (11.11)
	Sardasht	8	5 (62.50)	4 (50.00)
	Piranshahr	9	4 (0)	0 (0)
	Oshnavieh	16	4 (0)	0 (0)
	Chaldoran	5	0 (0)	0 (0)
	Salmas	11	4 (0)	1 (0)
	Khoy	4	0 (0)	0 (0)
	Showt	1	1 (100)	0 (0)
	**Total**	**107**	**23 (21.50)**	**6 (5.61)**

In Kurdistan province, among 130 collected water samples, 17 (13.08%) and 5 (3.85%) samples were positive for *ISFtu2* and *fopA* genes of *Francisella* spp., respectively. In West Azerbaijan province, 23 (21.50%) and 6 (5.61%) samples of the 107 collected water samples, were positive for *ISFtu2* and *fopA* genes of *Francisella* spp., respectively. There was no statistical significant difference in positivity of both genes between these two provinces (P>0.05).

## DISCUSSION

In this study, culture and also real-time PCR were used for examination of samples. The results demonstrated the contamination of natural water surfaces of the north western part of the country with *Francisella* spp. Only in the molecular tests, evidence was found for the presence of *F. tularensis* in the collected sample waters. The sequencing results indicated the presence of some other bacterial genera in water samples in the studied area.

*F. tularensis* subspecies *tularensis* is distributed in the USA, and very rare reports have been described elsewhere in the world. However, *F. tularensis* sub-species *holarctica* is reported as abundant species in Europe and Asia (2) and surface waters play an important role in the survival of bacteria in the nature. In some studies, the rate of contamination with *F. tularensis* in water sources is determined. In a survey in Sweden, 32% of water samples and 20% of the sediment of these sources were contaminated with *F. tularensis* subspecies *holarctica* (20). Results in another study that conducted in Massachusetts, USA, showed that none of the samples from marsh sites were contaminated with this agent but some of the brackish water samples were positive for 16S rDNA gene (21). In spring water sample in Utah USA, totally 39 *Francisella* spp. were isolated that from these samples, 79% belonged to *F. philomiragia* and 21% were *F. tularensis* ssp. *novicida* (22). The prevalence of *F. tularensis* contamination in soil samples at Lahore province in Pakistan, Iran southeastern neighbor, was 13.1% (23). In recent years, several major outbreaks of tularemia with the holarctica subtype and the source of water are reported in Turkey (24) adjacent to the studied provinces in our study.

Unfortunately, bacterial isolation was not possible in this study. It seems that use of laboratory animal inoculation could increase the chance to isolate the agent. In this study, 40 specimens were positive in real-time PCR molecular methods, which were negative in culture. The major and probable reason for culture negative and positive real-time PCR results might be due to viable but non-cultural state of this agent in the nature (25) or the introduction of a bacterium in aqueous environments within the amoeba as well as from the disappearance of the bacterial vegetative form in the water and the presence of the DNA sample in water (26). Some studies have shown that in a viable but non-culturable state, the virulence of this agent does not suffice to kill the mouse and, at the same time, is not able to grow on the experimental culture environments, but the metabolic pathways were completely active (25, 27).

In the case of *Legionella*, which has microbiological characteristics close to *Francisella*, such as the need for L-Cystein, intracellular persistence, survival in amoeba and water, it has been proven that the bacterium remains in the amoebas, and the use of amoebae as the basis of the culture medium, can greatly increase the chances of isolation of these agents from samples such as water samples (28). Information on the volubility of the use of amoebas as a culture medium for *Francisella* spp. has not been reported, but it seems an effective way to promote the isolation of intracellular bacteria. It was better if we used the special culture methods such as Amoeba co-culture in the water for cultivation which would permit further proliferation of the bacteria within the amoeba (base medium) and ultimately lysis of amoeba and culture of agent in a laboratory culture medium (29).

Given the geography of Iran, and the lack of reports of *F. tularensis* subspecies tularensis outside of North America, positive specimens in this study should belong to subspecies holarctica. Fortunately, these years almost all the village all over Iran have complete access to treated water and the water distribution network of our country have covered all the villages and so the human cases in the contaminated areas should be low. Fortunately, in this study all kind of surface water were selected to better investigate the *Francisella* spp. distribution status in the sampling locations. Limitations of this study was that only 2.5 liter of water were collected from each location and the collection season in the study was summer. It seems that the bigger amount of water sample and collection of samples in the late weeks of winter or the early weeks of spring could increase the chance of finding positive results in the culture.

## CONCLUSION

In a classic and standardized microbiological study, this study failed to isolate bacteria from collected natural surface water samples in the north western part of the country; however, the positive results by molecular methods indicate the contamination with *Francisella* spp. Clinical report of Tularemia in 1980 in Kurdistan province (6), recent epidemiological study in this region with high prevalence (11), finding of the antibody against tularemia in rodents in this province (9), and the molecular positive samples in this study all confirm the presence of bacterium in this region, which gives hopes in further experiments we will succeed also to isolate this agent from the environmental samples such as water, amoeba and ticks as well as the clinical samples.
